# 

**Published:** 1917-11-03

**Authors:** 


### CONTENTS.

| | PAGE |
|---|---|
| **On Certainties and Probabilities...** ... ... | 85 |
| **Thinking in Calories** ... ... ... ... ... | 86 |
| **Hospital and Institutional News** ... ... ... | 87 |
| The Treatment of Shell-shock—The Home of Recovery—The Death of Sir Pardey Lukis—Red Cross Aeroplanes—Special Constables' Gift of Ambulances—Civilian Doctors and the R.A.M.C.—Ozone for Wounds—An Undenominational "Chaplain" Suggested—Incurables' Hospital to Change its Name—Arundel Emergency Hospital—Danger in Swimming Baths—Kiss-in-the-Ring and Diphtheria—School Medical Services—The Children of the Mine-sweepers—The First Annual Report—Training the Interned—Excessive Prescribing in Derbyshire—Honours Won by So-called Criminals—Wages in Lieu of Holidays—The South Wales Nursing Association—A Gazetteless Hospital—The Birmingham Draughtsmen—The Fourth Southern *Gazette*—This Week's Drug Market—To Our Readers. | |
| **Anæsthetics in Military Surgery** ... ... | 93 |
| Nitrous Oxide and Oxygen. | |
| Advantages and Disadvantages. | |
| **The Organisation of Sectional Subscriptions** | 95 |
| The Way to Kill Waiting-Lists, and How to Pay for Additional Upkeep. | |
| **King Edward's Hospital Fund for London** ... | 96 |
| The Statistics of 109 Metropolitan Hospitals for 1916. | |
| **Practical Food of Real Value** ... ... ... | 97 |
| I. Why and Where Hospitals Cost More. | |
| **Parliament and the Profession** ... ... ... | 98 |
| **The Editor's Letter-Box** ... ... ... ... | 98 |
| "Raising a Ghost"—and Laying a Controversy. | |
| **The Institutional Library** ... .. ... ... | 99 |
| The Small Community Hospital—The Treatment of War Wounds—The Dietetic Treatment of Diabetes—Health of Working Girls—The Canteeners—Gold Stripes—Bread and Fancy Breads. | |
| **The Matrons' and Sisters' Department** ... | 101 |
| The College of Nursing, Limited. | |
| Round the Hospitals. | |
| British Nurses of the Best—The Prime Minister's Tribute—Their Newspaper Friends—The Nation's Fund for Nurses—Honour for Miss Amy Hughes—A Hospital as Profiteer. | |
| **Nursing Affairs in Ireland** ... .. ... ... | x |
| **The Institutional Worker** | l |
| Starting a Venereal Block. | |
| Questions for November. | |
| Enquiries and Answers. | |

